# Strong inhibitory activities and action modes of lipopeptides on lipase

**DOI:** 10.1080/14756366.2020.1734798

**Published:** 2020-03-27

**Authors:** Mei-chun Chen, Tian-tian Liu, Jie-ping Wang, Yan-ping Chen, Qing-xi Chen, Yu-jing Zhu, Bo Liu

**Affiliations:** a Agricultural Bioresources Research Institute, Fujian Academy of Agricultural Sciences, Fuzhou, China; b College of Biological Science and Engineering, Xiamen University, Xiamen, China

**Keywords:** Lipopeptides, inhibitory activities, lipase, action mode

## Abstract

Lipopeptides have been reported to exhibit anti-obesity effects. In this study, we obtained a *Bacillus velezensis* strain FJAT-52631 that could coproduce iturins, fengycins, and surfactins. Results showed that the FJAT-52631 crude lipopeptide, purified fengycin, iturin, and surfactin standards exhibited strong inhibition activities against lipase with dose-dependence manners (half maximal inhibitory concentration (IC_50_) = 0.011, 0.005, 0.056, and 0.005 mg/mL, respectively). Moreover, fengycin and surfactin had the comparable activities with orlistat, but iturin not. It was revealed that the inhibition mechanism and type of the lipopeptides were reversible and competitive. The quenching mechanism of lipase was static and only one binding site between lipase and lipopoeptide was inferred from the fluorescence analysis. The docking analysis displayed that fengycin and surfactin could directly interact with the active amino acid residues (Ser or Asp) of lipase, but not with iturin. Our work suggests that the *B. velezensis* lipopeptides would have great potential to act as lipase inhibitors.

## 1. Introduction

The incidence of obesity has increased with astounding rapidity worldwide, rendering obesity a serious public health concern of the 21st century. Obesity is a complex, multifactorial disease that arises from the interaction of excessive caloric intake, sedentary lifestyle, metabolic disorder, and genetic predisposition^1^. Indeed, obesity is the risk factor responsible for various chronic metabolic diseases or syndromes including diabetes mellitus, hypertension, hyperlipidaemia, osteoarthritis, hepatic steatosis, and so on^2^^,^^3^.

Dietary and lifestyle modifications such as calorie restriction and physical exercises are the common strategies adopted to control body weight; further, these methods have limited anti-obesity effects^4^. It has previously been reported that lipase inhibition is a potential strategy for counteracting obesity. Digestive lipase hydrolyses non-absorbable dietary triglycerides to smaller absorbable molecules of monoglycerides and free fatty acids, which are absorbed by the intestine. Inhibiting digestive lipase can reduce intestinal fat absorption^5–7^. Human pancreatic lipase is the main enzyme in intestinal digestion of dietary fats in the human digestive system. To date, a wide variety of natural products have been used as pancreatic lipase inhibitors, which originate from plants and metabolites of microorganisms. These include lipstatin, panclicins, saponins, polyphenols, flavonoids, caffeine, chitin, chitosan, etc^5^. The lipase inhibitor orlistat is the only one obesity-treatment drug currently available in the market, which reduces intestinal fat absorption via inhibition of pancreatic lipase; however, it has been reported to cause certain side effects, e.g. oily stools, oily spotting, and flatulence^1^^,^^8^. Some polyphenol compounds have been reported to have potential adverse effects on microorganisms and animal at high concentrations^9^^,^^10^. Thus, there is still a need to explore safe and effective anti-obesity drugs.

Interestingly, surfactants were found to produce inhibitory effects on the lipolytic efficiency of lipase by generating inactive aqueous enzyme-surfactant complexes or by blocking the congregation of enzymes at the lipid/water interface^11^. In fact, it has been reported that bacterial cyclic lipopeptides are the most popular amphiphilic molecules that are excellent surface active compounds^12^. The genus *Bacillus* is described as an efficient source of lipopeptide biosurfactants. The *Bacillus* lipopeptides are divided into three different families, including iturins, surfactins, and fengycins, consisting of a cyclic lipoheptapeptide or decapeptide with a long hydrophobic fatty acid moiety^13^. Surfactin is a well-known surfactant consisting of a peptide ring of seven amino acids with a β-hydroxy-fatty-acid chain that can lower the surface tension of water from 72 to 27 mN/m^14^. In contrast to surfactin, iturin contains a β-amino fatty acid linked to a peptide ring with seven amino acid residues, while fengycin is a cycle lipopeptide with 10 amino acid residues. It has been reported that the lipopeptide biosurfactants exhibit numerous bioactivities, such as antimicrobial, antiadhesive, antitumoral, antiviral, and hypoglycaemic activities^15–17^. Additionally, the *Bacillus* lipopeptides possess high biodegradability, biocompatibility, and high stability towards extreme environments. These remarkable properties make lipopeptides potent candidate drugs for therapeutic medical applications^18^.

It had been reported that lipopeptides of *Bacillus subtilis* SPB1 could significantly reduce the body weight of obese rats and relieve hyperlipidaemia without apparent side effects^18^^,^^19^. The anti-obesity effects are mediated by lipopeptides through inhibiting the serum pancreatic lipase activity to modulate dietary triglyceride digestion^18^^,^^19^. However, the molecular mechanism of lipopeptide interaction with lipase needs further exploration. The *B. subtilis* SPB1 lipopeptides consist of iturins, surfactins, fengycins, and other lipopeptide isoforms. Moreover, the surfactins were speculated to be the major contributor to the anti-obesity effects of *B. subtilis* SPB1 lipopeptide. However, it is still unknown whether all the types of lipopeptides display the inhibition effect on the lipase. Considering the structural differences between different lipopeptide families, the comparative studies of lipase inhibition activities of surfactin, iturin, and fengycin would be important for the application of lipopeptide as lipase inhibitor. The aim of this article is to report a new lipopeptide-produced *Bacillus velezensis* strain FJAT-52631 that could coproduce iturin, fengycin, and surfactin and to evaluate the inhibition activity of each type of lipopeptide. Furthermore, the action modes of lipopeptide on lipase catalysis was carried out.

## 2. Materials and methods

### 2.1. Chemicals and strains

Lyophilised powder of *Mucor miehei* lipase (EC3.1.1.3), 4-Nitrophenyl palmitate (4-NPP), iturin, and surfactin were purchased from Sigma-Aldrich (St. Louis, MO). Acetonitrile, hydrochloric acid (HCl) and Tris were purchased from Sinopharm (Shanghai, China).

The strain FJAT-52631 (CCTCC No. M 2019760) was isolated from a soil sample from Wuyi Mountain, Fujian Province, China and it was identified through whole genome sequence analyses.

### 2.2. Lipopeptide extraction and preparation

A single clone of the strain FJAT-52631 was inoculated in a 25-ml sterile tube with 5 ml liquid culture media (beef Extract 3 g/L, peptone 5 g/L, and glucose 10 g/L) and incubated for 25 h at 30 °C and 170 rpm. The pre-culture was inoculated (1%) into 250-mL flasks with a 50 mL potato dextrose broth culture medium and then cultivated for 48 h in a rotary shaker at 30 °C, 170 rpm. After fermentation, the cells were removed by centrifugation (6000 g for 5 min) and the lipopeptide in the culture supernatant was precipitated by adding 3 N HCl to achieve a final pH of 2. The precipitates were dissolved in a phosphate buffer and then lyophilised for anti-lipase activity tests and liquid chromatography quadrupole time-of-flight tandem mass spectrometry (LC-QTOF-MS/MS) analyses.

### 2.3. Lipopeptide identification and separation

The qualitative and quantitative analyses of lipopeptides produced from the FJAT-52631 were carried out using the LC-QTOF-MS/MS method described in our previous studies^20^. Then, the lipopeptides were purified using the C_18_ solid phase extraction method with methanol/water (v/v) as an elution solvent. Each elution fraction was evaporated at a reduced pressure (−50 psig, 50 °C), dissolved in water and then lyophilised.

### 2.4. Measurement of lipase activity

The lipase inhibition was determined according to the method described by Liu et al.^21^ 10 µg/mL lipase in water and 7.5 mmol/L 4-NPP in acetonitrile solutions were prepared. The crude lipopeptide and purified fengycin were dissolved in water, while the iturin and surfactin standards were dissolved in methanol, and then all were diluted to their appropriate concentrations. The 1 mL reaction mixture contained 0.75 mM 4-NPP, 0.4 µg/mL lipase, and different concentrations of inhibitor in Tris-HCl buffer (pH 7.8). The reaction was carried out at 37 °C and the detection wavelength was set at 405 nm.

The inhibition mechanisms were studied by fixing the concentration of substrate and changing the lipopeptides and enzymes to monitor enzymatic reaction. The inhibition types were determined based on the Lineweaver-Burk plot^22^; this reaction system contained different concentrations of substrate and lipopeptide and 100 µL of lipase in Tris-HCl buffer. Then, the inhibition constant was calculated from a secondary plot of 1/V_m_ versus the inhibitor concentration.

### 2.5. Spectrum analysis

Ultraviolet (UV) wavelength scanning spectra of the 4-NPP hydrolysis product was measured in the absence and in the presence of lipopeptide using a model 1510 spectrophotometer (Thermo Fischer Scientific, Waltham, MA). The 1 mL reaction mixture contained 0.75 mmol/L 4-NPP and 0.015 mg/mL of lipopeptide in Tris-HCl buffer (pH 7.8). The final concentration of the lipase was 0.4 µg/mL.

Fluorescence quenching spectra of the lipase and the lipopeptide were carried out according to the method described by Liu et al.^21^, using a Cary Eclipse spectrophotometer (Agilent, Santa Clara, CA). The Stern-Volmer quenching constant (K_sv_) was calculated according to Equation (1):

(1) $$F_{0}=1+{\text{K}}_{\text{sv}}[l],$$

where *F* and *F*_0_ are the fluorescence intensities with and without lipopeptide, respectively; further, [*I*] is the concentration of the lipopeptide. The binding constant (K_A_) and the binding affinity (*n*) were calculated according to the Equation (2):

(2) $$\text{lg}[(F_{0}-F)/F]={\text{IgK}}_{\text{A}}+n\text{lg}[l].$$

### 2.6. *In silico* simulating molecular docking model

The interactions between lipopeptide and lipase were modelled using molecular operation environment software (MOE). The energy between the lipase and the ligand was minimised before docking. The docking parameters were set according to a previous study^21^.

## 3. Results and discussion

### 3.1. Identification of the strain FJAT-52631

A lipopeptide-produced strain FJAT-52631 was isolated from the soil sample, which was further accurately identified through the whole genome sequence analyses. The genome sequence of strain FJAT-52631 contains 3929791 bp (GenBank accession number: CP045186). The G + C content of the chromosomal DNA for strains FJAT-52631 was 46.5 mol %. The genome-based similarity calculated based on the OrthoANIu between strain FJAT-52631 and the type strain *Bacillus velezensis* CBMB205^T^ was 99.95%. This value was above the threshold ANI value of 95–96% used for delineating prokaryotic species, suggesting that strain FJAT-52631 is a strain of the species *B. velezensis*^23^.

### 3.2. Identification and separation of lipopeptides produced by the strain *B. velezensis* FJAT-52631

The lipopeptides produced from the strain FJAT-52631 were identified using the LC-QTOF-MS/MS method. Results revealed that three sets of homologue molecules with retention times in the range of 12.2–21.5, 27.0–38.7, and 45.2–54.0 min could be categorised as iturins, fengycins, and surfactins, respectively. Peptide sequences of each lipopeptide group were determined based on previous literature reports^24–26^. The retention times, MS and MS^2^ spectral data, and identification results were summarised, as shown in Table 1; the iturins consisted of C_14_–C_16_ iturin A; the surfactins consisted of C_12_–C_16_ surfactin A, and C_16_ surfactin A derivative; further, the fengycins consisted of C_16_/C_18_ fengycin A, C_16_ fengycin A_2_/B_2_, C_16_–C_17_ fengycin B, and C_15_ fengycin A/B derivatives. The iturin, fengycin, and surfactin content in the supernatant were calculated as 2.66 ± 1.50, 86.95 ± 4.08, and 35.93 ± 2.28 mg/L, respectively. The results demonstrated that fengycin was the most abundant lipopeptide family that was produced by the strain FJAT-52631.The lipopeptide production from *Bacillus* spp. firstly depends on itself. For example, the *B. subtilis* SPB1 strain have the ability to coproduce iturins, fengycins, and surfactins and highest content of surfactin in lipopeptide mixture was observed^15^.

**Table 1.** Identification of lipopeptides produced by *B. velezensis* FJAT-52631 using LC-QTOF-MS/MS.

RT	MS *m/z* [M + H]^+^/[M + Na]^+^	MS^2^*m/z* [M + H]^+^/[M + Na]^+^	Identification of lipopeptides
*Iturin*			
12.221	1065.5	1049.5,1038.5,1021.5,1007.5, 955.5, 934.5, 796.4, 740.4, 656.4, 497.2, 378.1	C_14_ iturin A
15.295	1079.5	1062.5, 1051.5, 1034.5, 1020.5, 992.5, 968.5, 947.5, 497.1, 378.1	C_15_ iturin A
15.837	1079.5	1062.5,1051.5,1034.5,1021.5, 968.5, 639.2,378.1	C_15_ iturin A
16.741	1079.6	1062.5,1051.6, 1034.5,1021.5, 968.5, 736.4,384.1	C_15_ iturin A
20.810	1093.6	1077.5, 1066.6, 1049.6, 544.3, 382.2	C_16_ iturin A
21.443	1093.6	1077.5, 1066.6, 1049.6, 544.3, 382.2	C_16_ iturin A
*Fengycin*			
30.892	1449.8	1088.5, 974.4, 664.3, 463.1,	C_16_fengycinA_2_
32.565	1463.8	1102.5, 988.5, 678.3, 581.2, 463.1, 389.2	C_16_ fengycinA
33.333	1477.9	1116.6, 1002.5, 692.3, 491.2	C_16_ fengycinB_2_
34.509	1491.9	1130.6, 1016.5, 706.4, 485.3	C_16_ fengycinB
36.227	1505.9	1130.5, 1016.5, 895.4, 620.3	C_17_ fengycinB
37.221	1447.8	1102.5, 988.5, 678.3, 485.3	C_15_ fengycinA derivative^a^
37.583	1491.8	1102.6, 988.5, 565.3, 454.2	C_18_ fengycinA
38.713	1475.8	1130.6, 1016.6, 593.3, 423.2	C_15_ fengycinB derivative^a^
*Surfactin*			
45.258	1016.7	1014.6, 945.6, 832.5, 742.5, 594.4, 498.3, 391.3	C_12_ surfactin A
45.755	1016.7	1014.6, 950.6, 836.5, 746.5, 594.4, 481.3, 391.3	C_12_ surfactin A
46.433	1016.6	1016.6, 903.6, 790.5, 700.5, 594.4, 481.3, 391.3	C_12_ surfactin A
47.156	1076.7	1028.6, 964.6, 915.5, 851.5, 761.5, 594.4, 498.3	C_16_ surfactin A derivative
47.925	1030.6	917.6, 804.5, 714.5, 643.5, 594.4, 481.3	C_13_ surfactin A
49.824	1044.7	931.6, 818.5, 728.5, 657.5, 594.4, 481.3, 391.3	C_14_ surfactin A
50.276	1044.7	931.6, 818.5, 728.5, 657.5, 594.4, 481.3, 391.3	C_14_ surfactin A
51.542	1058.7	945.6, 832.5, 742.5, 671.5, 594.4, 481.3, 391.3	C_15_ surfactin A
52.446	1058.6	945.6, 832.5, 742.5, 671.5, 594.4, 481.3, 391.3	C_15_ surfactin A
53.124	1072.7	960.6, 847.6, 752.5, 712.5, 594.5, 481.3, 391.3	C_16_ surfactin A
53.667	1072.7	959.6, 846.6, 756.6, 685.5, 594.4, 481.3	C_16_ surfactin A

^a^Monounsaturated β-hydroxy fatty acid.

**Figure 1.**
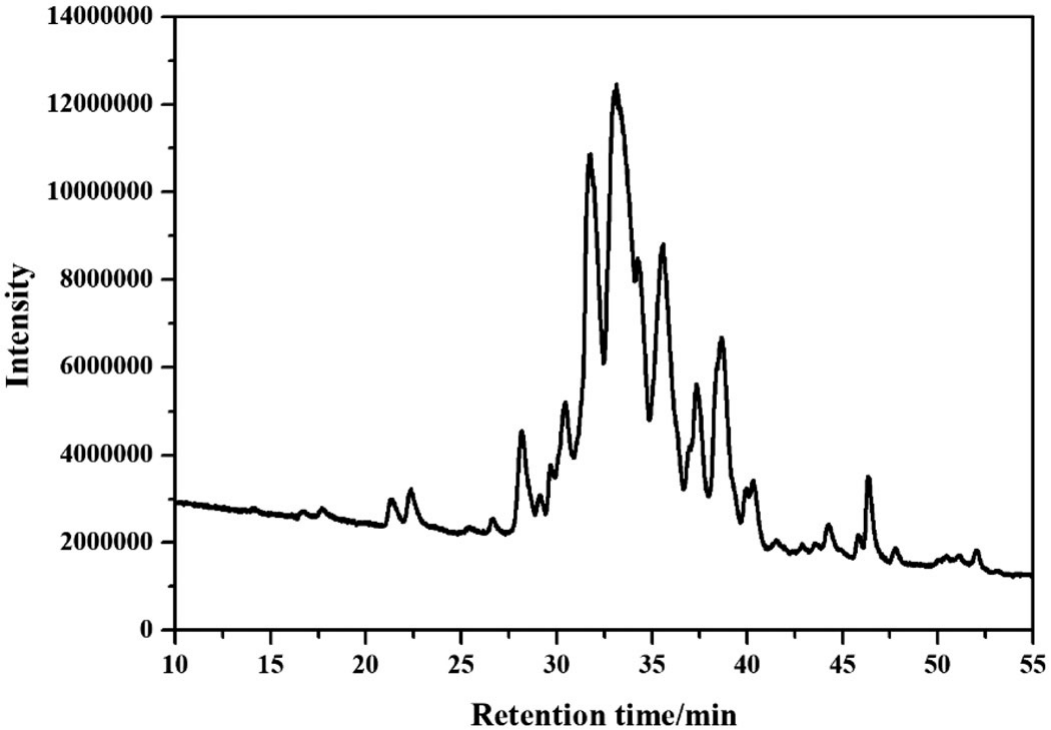
The full scan LC-ESI-MS chromatogram of fraction eluted using 80% methanol.

**Figure 2.**
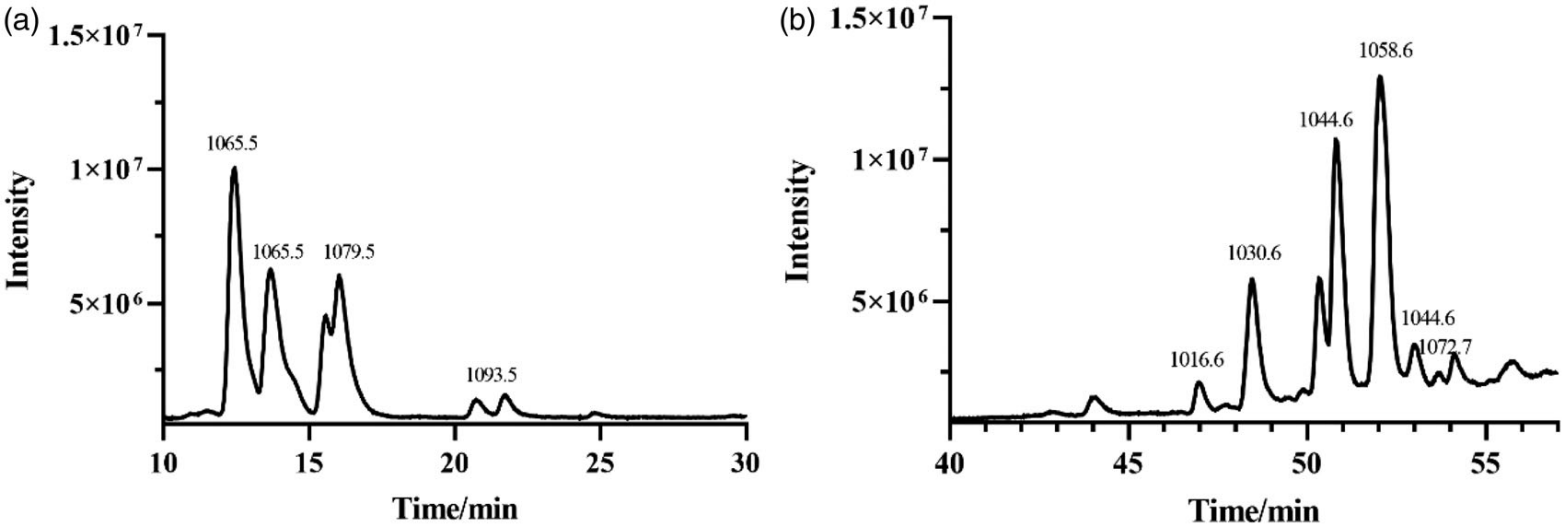
The full scan LC-ESI-MS chromatogram of (a) iturin and (b) surfactin standards.

The crude lipopeptide from strain FJAT-52631 was further purified by solid-phase extraction method, and characterised by LC-TOF-MS analysis. Results show that the purified fengycin in the crude lipopeptide was obtained by elution with 80% methanol (Figure 1), while the iturin and surfactin in the crude lipopeptide were not successfully purified using the above method. LC-QTOF-MS analysis revealed that the composition of iturin and surfactin in the crude lipopeptide yield from FJAT-52631 were identical to the iturin and surfactin standards (Figure 2). Thus, iturin and surfactin standards were used to substitute the corresponding substances in further study.

### 3.3. Inhibitory activity of lipopeptide acting on lipase

**Figure 3.**
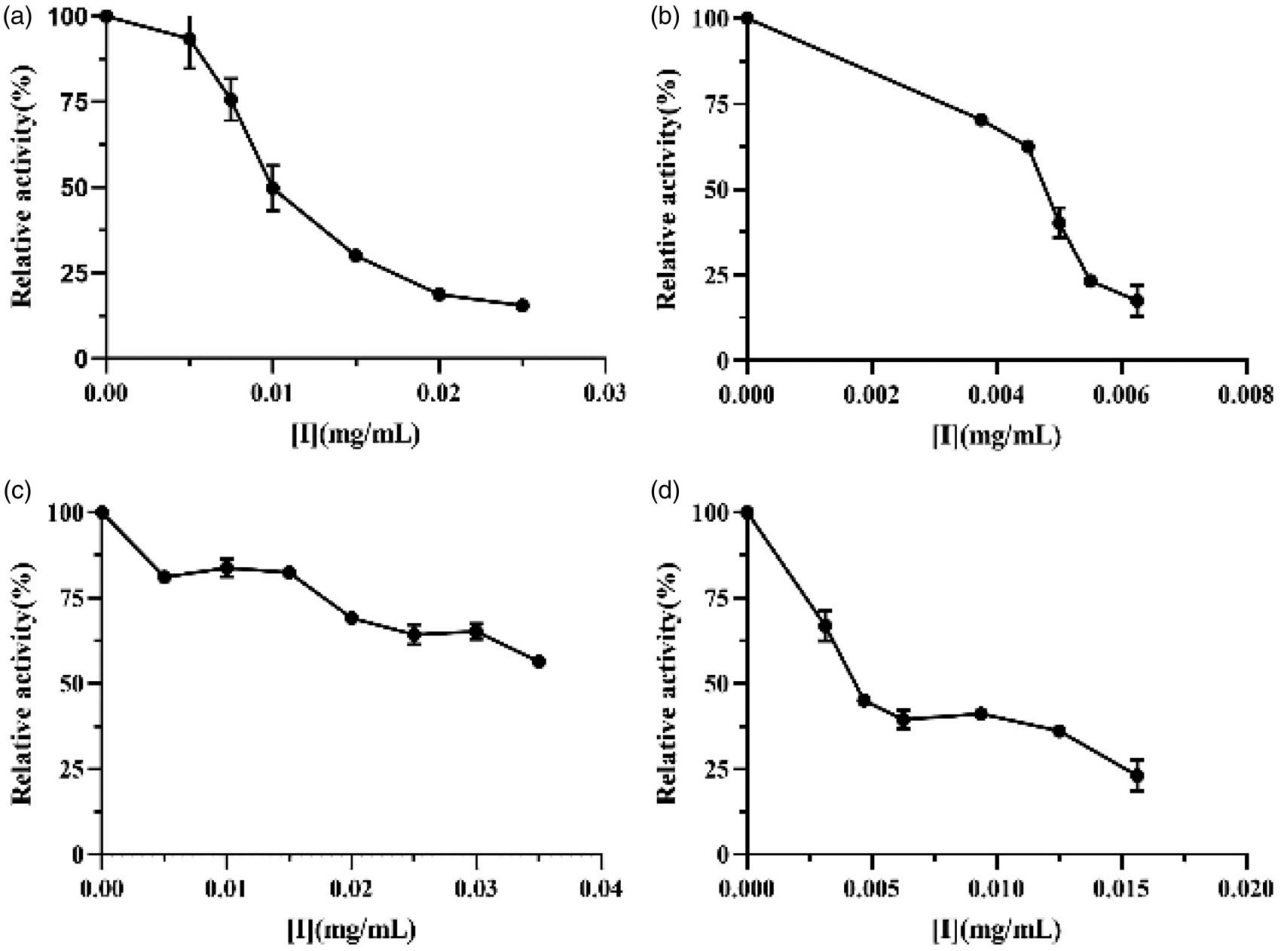
Lipase inhibitory activity of (a) crude lipopeptide, (b) purified fengycin, (c) iturin, and (d) surfactin.

As reported in literatures, surfactants could inhibit lipase activity at certain concentrations due to their special property^14^. Lipopeptides from the *Bacillus* group have been proved to be excellent surfactants and could reduce lipase activity in plasma from alloxan-induced diabetic rats^18^. In spite of this, the manner of lipopeptide interaction with lipase and the mechanism of enzyme inhibition are still unknown. In the present study, we report the effects of crude lipopeptide (Figure 3(a)), purified fengycin (Figure 3(b)), iturin (Figure 3(c)), and surfactin (Figure 3(d)) standards on the lipase. Both the lipase from *M. miehei* and human pancreatic lipase are serine proteases, which possess the same active site and catalytic mode^27^. Moreover, the lipase from *M. miehei* exhibits much higher enzyme activity, better purity, and more mature processes than the human pancreatic lipase^28^. Thus, the *M. miehei* lipase was selected for use in the present study.

Results showed that lipase inhibition was dose dependent. After adding these four inhibitors, the relative enzyme activity decreased significantly with increasing concentration of effectors. The half maximal inhibitory concentration (IC_50_) of crude lipopeptide, purified fengycin, iturin, and surfactin standards were 0.011, 0.005, 0.056, and 0.005 mg/mL, respectively. We have previously reported that furoic acid and oxalic acid inhibited lipase with IC_50_ of 0.242 and 1.425 mg/mL, respectively^21^. Moreover, we found that the inhibitory effects of the lipopeptides were within one order of magnitude of orlistat (IC_50_ = 0.004 mg/mL)^7^. These results suggest that lipopeptides have strong lipase inhibition activity and could act as lipase inhibitors to prevent obesity.

The anti-lipase activities of natural compounds depend on their structure characteristics, including number and position of hydroxyl groups, degree of polymerisation, elimination of glycosylation, and the size of the molecules^6^. Buchholz et al.^6^have reported that a high number of phenolic hydroxyl groups in active flavonoids increases their inhibitory effect. In present study, it was found that the fengycin and surfactin exhibit much stronger inhibition activities on lipase (about 10-fold) than that of iturin, which possibly be attributed to the fengycins and surfactins composing of β-hydroxy fatty acids, whereas the iturins carrying a β-amino fatty acid modification^14^^,^^15^. The crude lipopeptide produced by FJAT-52631 was composed of 2.7% iturin, 73.9% fengycin, and 23.4% surfactin, which indicated that the inhibition effect of crude lipopeptide produced by FJAT-52631 was mainly attribute to the fengycin. To our knowledge, this is the first report of the strong inhibitory activity of lipopeptide from *B. velezensis* against lipase *in vitro*.

### 3.4. Inhibition mechanism and type of lipopeptide on lipase activity

**Figure 4.**
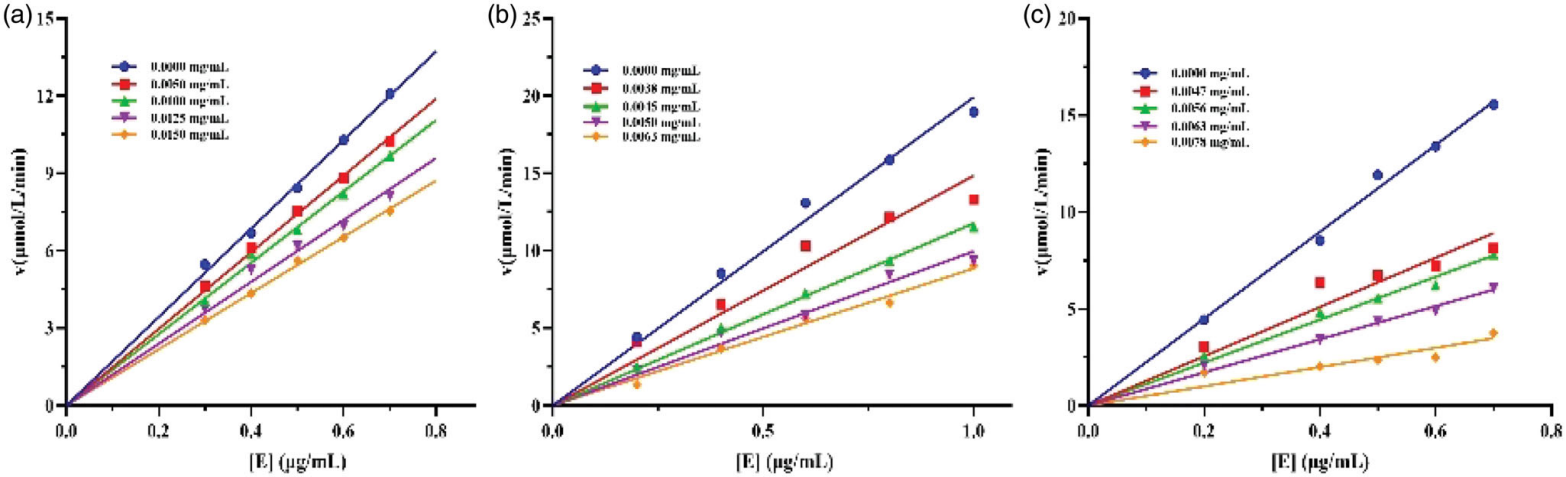
Inhibition mechanisms of (a) crude lipopeptide, (b) fengycin, and (c) surfactin on lipase.

The mechanism of enzyme inhibition by drugs can be either reversible or irreversible^29^. The inhibition mechanisms of crude lipopeptide, fengycin, and surfactin on lipase catalysis were studied. As shown in Figure 4, all the plots of the remaining enzyme activity versus the enzyme concentrations generated a series of lines intersecting at the origin, and whose slopes decreased with increasing lipopeptide content. These results demonstrated that the above catalytic action is reversible, which means that the lipopeptide declined the catalytic activity of lipase but did not lead to enzyme deactivation. Studies showed that most common mechanism of the inhibitor observed is reversible, such as furoic acid and oxalic acid, others like orlistat display irreversible effects^21^^,^^29^.

**Figure 5.**
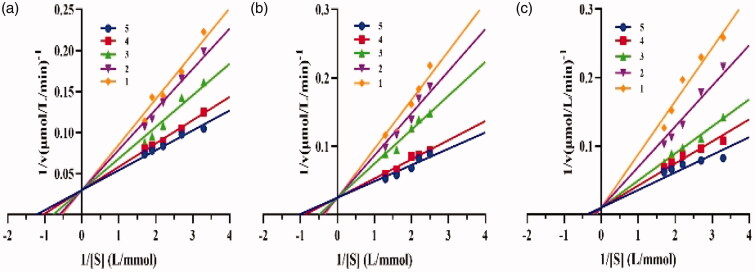
Inhibition types of (a), crude lipopeptide, line 5-1: mean 0, 0.0013, 0.002, 0.0025, 0.003 mg/mL, respectively; (b) fengycin, line 5-1: mean 0, 0.0013, 0.002, 0.0025, 0.003 mg/mL, respectively; and (c) surfactin, line 5-1: mean 0, 0.0008, 0.0009, 0.0011, 0.0013 mg/mL, respectively on lipase.

Four inhibition methods have been proposed for enzyme inhibitors, including competitive, non-competitive, uncompetitive, and mixed type^30^. The inhibition type could be related to the properties of inhibitors, such as structure, molecular weight, etc.^21^. To determine the inhibition type of crude lipopeptide, fengycin, and surfactin on lipase catalysis, plots of 1/*v* versus 1/[*S*] of lipopeptide on lipase shown in Figure 5 were drawn, where *v* is the reaction rate and *S* is the substrate concentration. The inhibition constants *K*_I_ of different lipopeptides were calculated and summarised in Table 2. Our results demonstrated that all the tested lipopeptides (mixture, fengycin, or surfactin) inhibit lipase in a competitive manner with respect to substrate concentration, which is subvert the explanation reported by Zouari et al.^18^, who deduced that *B. subtilis* SPB1 lipopeptide biosurfactant exhibits an uncompetitive inhibition mode action on lipase activity. Our above finding suggested that the lipopeptide binds to the free enzyme at the substrate-binding site.

**Table 2.** Inhibition effects of lipopeptides for lipase.

Compounds	IC_50_ (mg/mL)		Inhibition effect		Inhibition constant (mg/mL)
	Measured	Calculated	Mechanisms	Type	K_I_
Crude lipopeptide	0.011	0.008	Reversible	Competitive inhibition	0.003
Fengycin	0.005	0.002	Reversible	Competitive inhibition	0.001
Surfactin	0.005	0.001	Reversible	Competitive inhibition	0.001

### 3.5. Spectrum analysis of product in the presence and absence of lipopeptide

**Figure 6.**
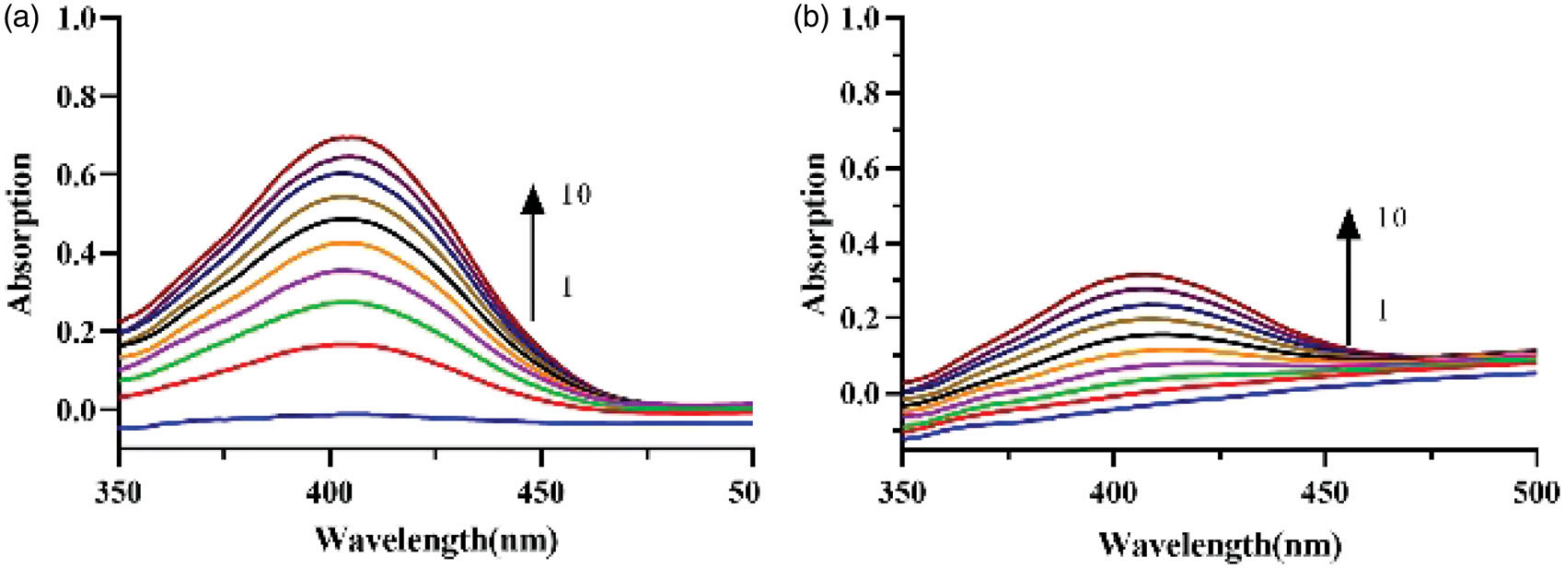
Consecutive spectra obtained during the lipase catalysis in the (a) absence and (b) presence of crude lipopeptide. Curves 1–10 depict the addition of the enzyme in 10 min.

The UV spectra of lipase catalysis in the absence (Figure 6(a)) and presence (Figure 6(b)) of the crude lipopeptide were measured. Results show that the typical peak absorbent intensity of the product at 405 nm increased along with an extension of the catalytic reaction time. After 10 min, in the presence of 0.015 mg/mL lipopeptide, the peak absorbance was reduced to 54.5%, which indicates that the crude lipopeptide produced from FJAT-52631 was a stronger inhibitor. This result was consistent with the enzyme activity assay.

**Figure 7.**
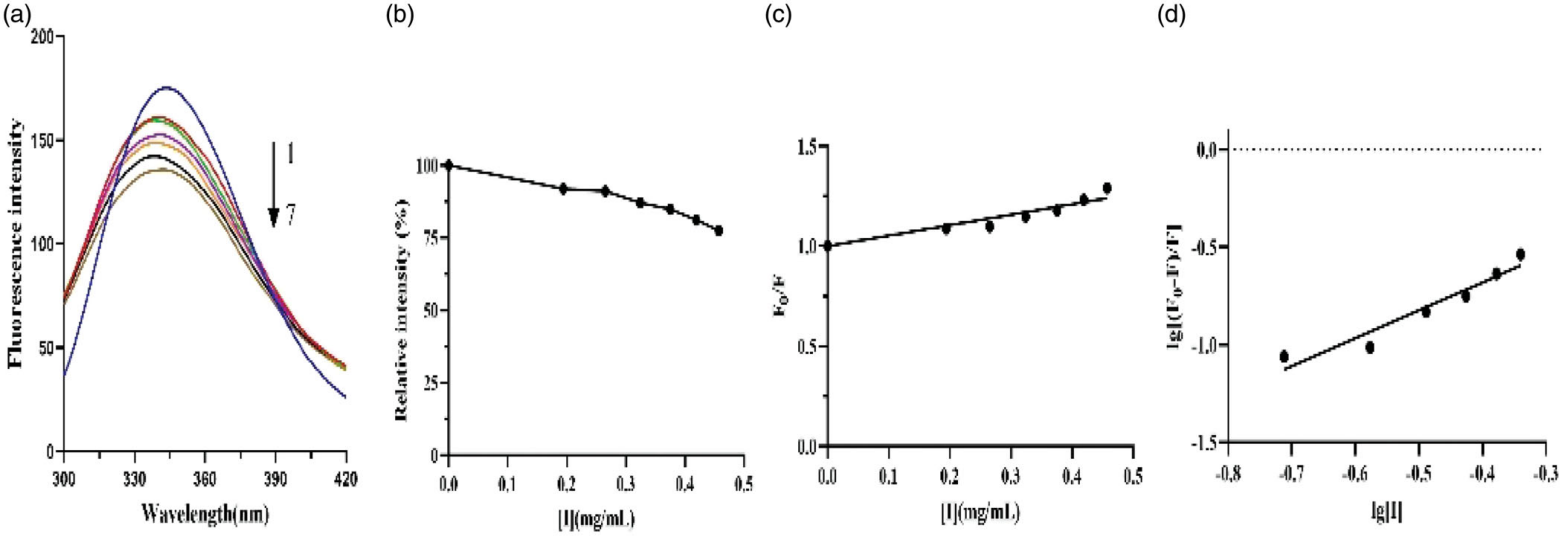
Effect of lipopeptides on the emission spectrum of lipase. (a) Emission spectra of lipase with crude lipopeptide concentration of 0, 0.194, 0.265, 0.324, 0.375, 0.419, and 0.457 mg/mL, respectively (1–7, respectively); (b) Florescence intensity changes with lipopeptide; (c) Plot of *F*_0_−*F* against [*I*] for lipopeptide; (d) Plot of lg[(*F*_0_−*F*)/*F*] versus lg[*I*] for lipopeptide.

The fluorescence emission spectra of crude lipopeptides on lipase are shown in Figure 7. Results showed that the fluorescence intensities of the emission peak at 338 nm gradually declined with increasing lipopeptide content (Figure 7(a,b)); this indicated that the lipopeptide and the lipase form a complex^21^^,^^31^. The effects of different concentrations of lipopeptides on lipase did not lead the endogenous fluorescence peak to red shift or blue shift; this suggests that there has not been a complete change of the composition of the enzyme molecule and that the enzyme is still active^32^. The plot of *F*_0_/*F* versus [*I*] was drawn and a linear regression equation of *F*_0_/*F* = 1 + 0.525[*I*] was obtained (Figure 7(c)). The value of Stern-Volmer quenching constant K_sv_ was found to be 0.525 L/g according to the Stern-Volmer equation. This value is larger than the maximum dynamic quenching constant of biomacromolecule (100 L/mol); this indicated that the fluorescence quenching mechanism of lipase is static when the lipopeptide is present^33^. Further, a linear regression equation lg[(*F*_0_−F)/*F*] = 1.429lg[*I*] − 0.108 (*R*^2^=0.9255) was obtained from Figure 7(d). The values of the binding constant K_A_ and binding site *n* from the intercept of the above equation and the slope were calculated as 1.429 L/g and 0.78, respectively. This result indicates that the interaction between lipase and lipopeptides is quite intensive and there is only one binding site between them^33^.

### 3.6. Molecular docking analysis

**Figure 8.**
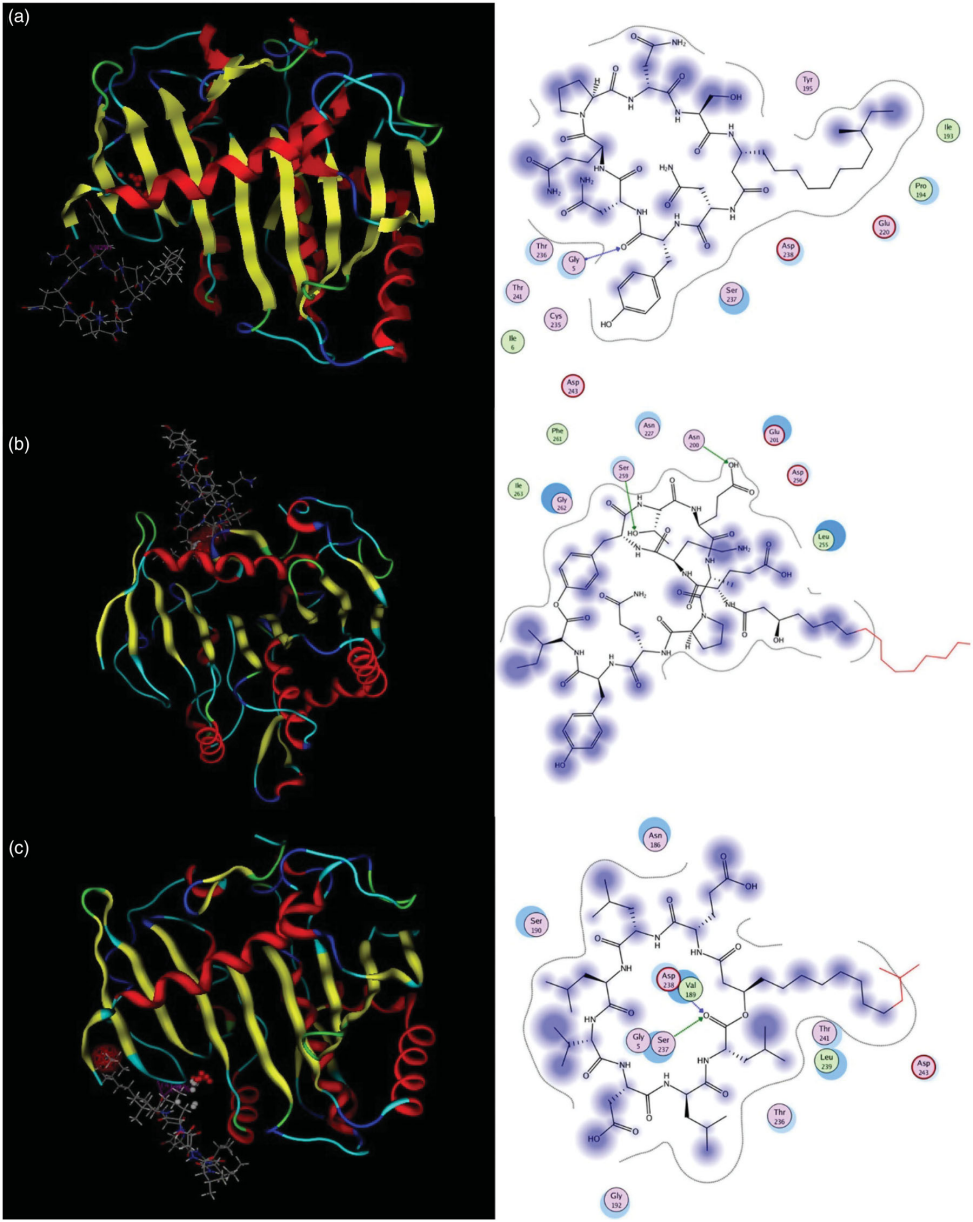
Interactions between key amino acids in (a) lipase and iturin, (b) fengycin, or (c) surfactin were investigated by in *silico* modelling.

The *M. miehei* lipase have three active site residues Ser, His, and Asp that forming an oxygen negative ion hole, which are covered by an “alpha-screw lid”^34^^,^^35^. The lipase was composed of hydrophilic groups surrounded by exposed hydrophobic and its activity depends upon the conformation of lid (in open or in closed)^35^. The conformation of lipase would be altered when the active site of lipase interacted with an inhibitor, leading to the reduced catalytic activity of lipase^36^. Previous studies have shown that the ring structure and the carbonyl groups of the inhibitor is important for their correct binding in the active site of lipase^37^. The molecular docking analysis was carried out to explore the possible binding action mode between lipopeptide and lipase. Results demonstrated that the carbonyl group on the Tyr residue in iturin A might directly act on Gly 5 of lipase (Figure 8(a)); the carbonyl group on the Leu residue in surfactin A might directly act on Asp 238 and Ser 237 of lipase (Figure 8(c)); and the oxygen atoms of the hydroxyl group on the Thr and Glu residues in fengycin might directly act on Ser 259 and Asn 100 of lipase (Figure 8(b)), respectively. The hydroxyl and carbonyl groups on the lipopeptides formed hydrogen bonds with the amino residues of lipase to stabilise the interactions between the lipopeptide and the lipase. This manner could block substrate access to the catalytic active site of the lipase enzyme. Our results displayed that the fengycin and surfactin could be in direct contact with the active amino acids of lipase, and not those of iturin. The interactions of oxygen with the catalytic site might enhance the lipase inhibition activity. Moreover, the active site residue Ser is essential for the hydrolytic activity of the enzyme^6^. This could explain in part the weaker potency of iturin. The above modelling results support the data derived from the enzymology studies.

Studies showed that the small molecules are easily to enter the gap in the oxygen negative ion to interact with the catalytic site of the enzyme. For example, the furoic acid and oxalic acid can directly act on active amino acid of Ser, while the big molecular orlistat does not contact with the active amino acids of, Ser, His or Asp^21^. Lipopeptides are the famous surfactants that composed of both hydrophilic and hydrophobic groups. The structures of surfactants play an important role in protein–surfactant interactions^35^. This suggested that lipopeptides lead to strong inhibition of *M. miehei* lipase activity by direct interact with the active site, which are closely related to the amphiphilic molecular structures of lipopeptides.

## 4. Conclusion

We report here on the strong inhibitory activity and action modes of lipopeptide from *B. velezensis* against lipase *in vitro*. The lipopeptides produced from *B. velezensis* FJAT-52631 were categorised as iturin, fengycin, and surfactin. The crude lipopeptides, iturin, fengycin, and surfactin exhibited strong inhibition activities against lipase with dose-dependence. The lipopeptide inhibition catalytic reaction was reversible and the inhibition type was competitive. The fengycins and surfactins directly interact with the residues of the enzyme’s active centre, while iturins do not. Fengycins were the most abundant compounds among the crude lipopeptides, which were considered to contribute significantly to the inhibited activities against lipase. Our results suggest that these lipopeptides could be used directly as lipase inhibitors and the *in vivo* anti-obesity effects of lipopeptides will be explored in further studies.
